# Supplementary figures and images for: Fucosyltransferase 8 (FUT8) and core fucose expression in oxidative stress response
Source: PLoS One. 2023 Feb 13;18(2):e0281516. doi: 10.1371/journal.pone.0281516 (PMC9924996; doi:10.1371/journal.pone.0281516)

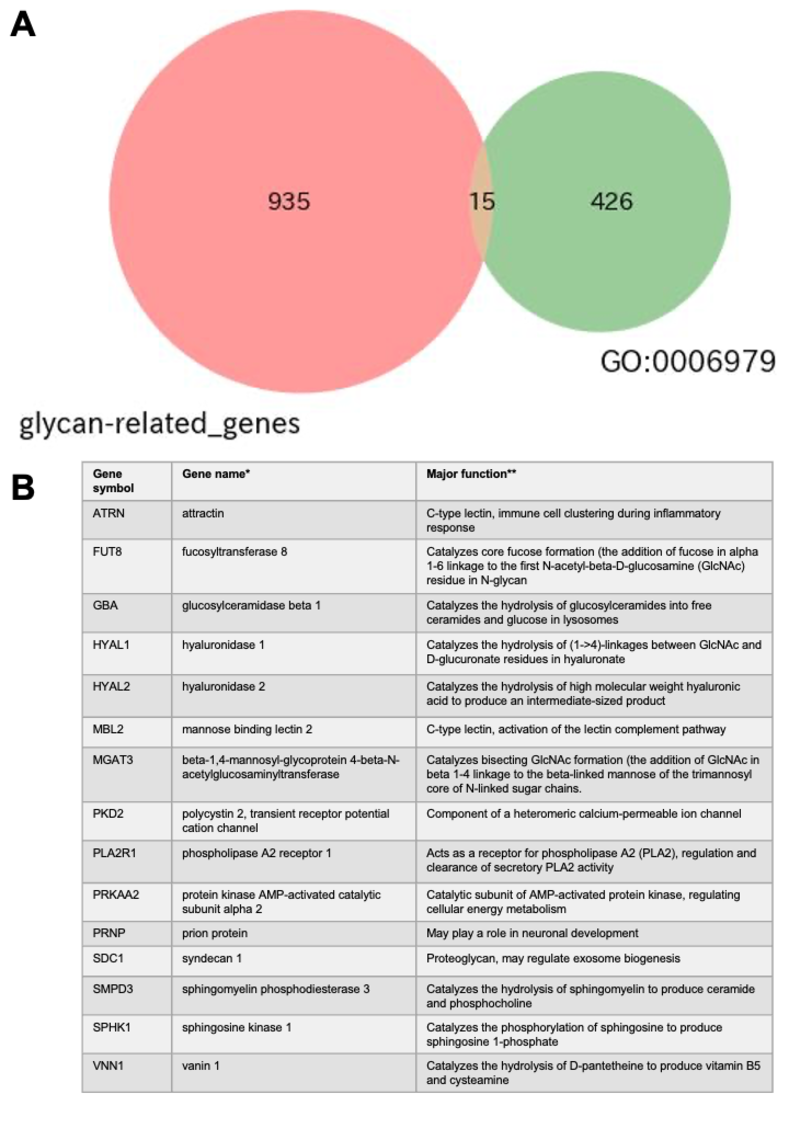

Supplement: S1 Fig — The antioxidant response defined by the Gene Ontology (GO:0006979 response to oxidative stress) includes 441 human genes, whereas GlycoMaple uses 950 human genes responsible for the biosynthesis of glycans (shown as glycan-related_genes). A) Fifteen genes overlap between these two sets, which are supposed to function in both antioxidant response and glycosylation. These genes are here called the 15 common genes, namely, ATRN, FUT8, GBA, HYAL1, HYAL2, MBL2, MGAT3, PKD2, PLA2R1, PRKAA2, PRNP, SDC1, SMPD3, SPHK1, and VNN1. Their gene names from geneames.org and their major functions from uniprot.org are summarized in B. (TIF) [file pone.0281516.s001.tif]

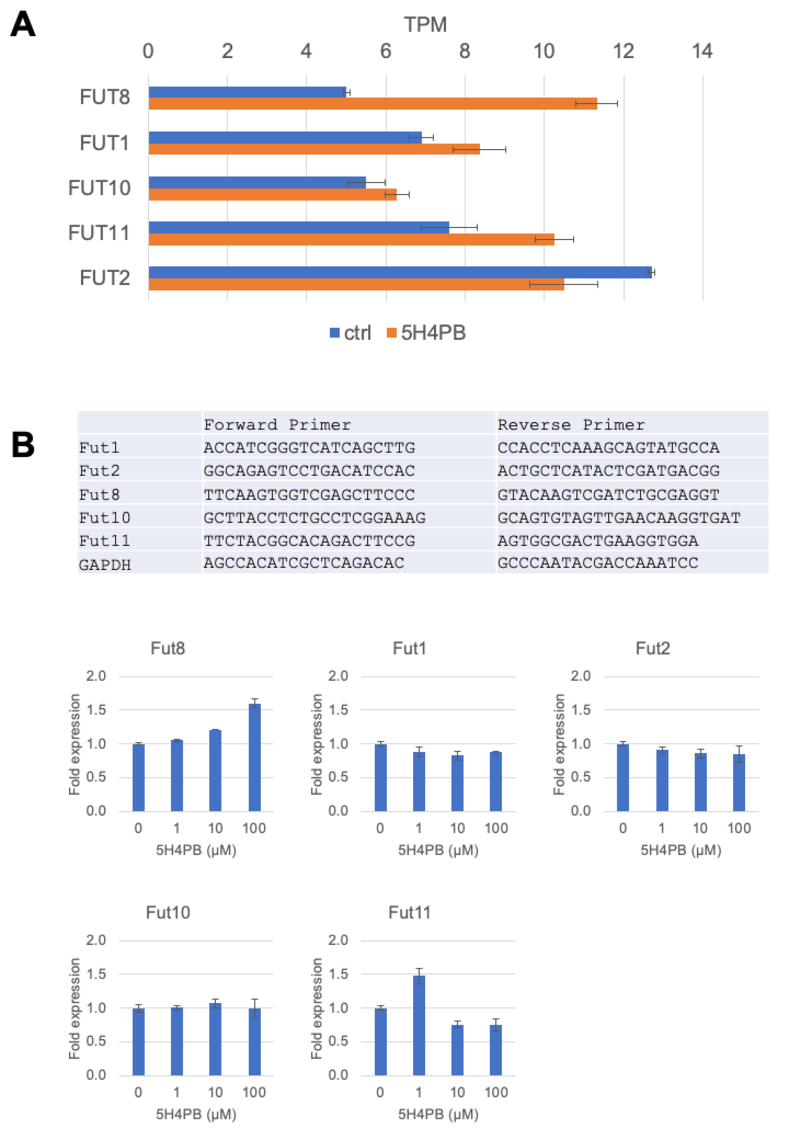

Supplement: S2 Fig — A) TPMs of fucosyltransferases expressed by the HaCaT cell line. Bar, S.D. (n = 2–3). *P<0.002. B) Relative mRNA expression of fucosyltransferases expressed by the HaCaT cell line. HaCaT cells were treated with the indicated concentration of 5H4PB for 24 h. Total RNA extraction, cDNA synthesis, and qPCR were performed using SuperPrep® II Cell Lysis & RT Kit for qPCR (SCQ-401; Toyobo, Tokyo, Japan). Sequences of primer sets used in this study are listed below. mRNA expression of fucosyltransferases was normalized by that of GAPDH. Fold expression of the indicated fucosyltransferases was calculated with that at 0 μM 5H4PB set as 1. Bar, S.D. (n = 2–3). (TIF) [file pone.0281516.s002.tif]
