# Supplementary material for: Fucosyltransferase 8 (FUT8) and core fucose expression in oxidative stress response
Source: PLoS One. 2023 Feb 13;18(2):e0281516. doi: 10.1371/journal.pone.0281516 (PMC9924996; doi:10.1371/journal.pone.0281516)
Supplement: Attachment — Submitted filename: rebut_ed.docx [file pone.0281516.s003.docx]

Reviewer #1: The manuscript reported the relationship between changes in glycosylation and oxidative stress. The authors found that FUT8, which encodes a core fucosyltransferase, was increased by treatment with antioxidant reagents, 5H4PB and SFN. The authors analyzed the changes of glycan structures by the GlycoMaple tool and lectin staining. However, the reviewer wonders whether the data sufficiently support the conclusions. The change of fucosylation was detected by UEA-I, whereas the UEA-I is not suitable for detection of the core fucose residue. The data obtained from the flow cytometric analysis should be quantified and analyzed statistically.

Our response: We performed the statistical analysis on the flow cytometric data and added the corresponding description and explanation of how we concluded that the increase in UEA-I signal is due to the increase in core fucose in the revised result section.

Although the upregulation of FUT8 was only detected by the genome-wide RNA-seq analysis, it is not confirmed by the individual quantitative PCR.

Our response: Our RNA-seq analysis detected FUT1, FUT2, FUT8, FUT10, and FUT11 among the enzymes responsible for fucosylation. Although FUT8 is the only enzyme whose fold change was significant, we confirmed that by qPCR, shown in Supplemental Figure S2, and added the corresponding explanation in the revised result section.

Without revising these points, it would be difficult to reach the levels of publication in PLOS ONE.

Our response: We hope that the changes above will meet the reviewer's requests and PLOS ONE requirements.

Reviewer #2: PONE-D-22-18338

This work is interesting and I recommend to accept it after the following modifications:

1.A detailed description of Flow cytometry methods should be provided.

Our response: We added this in the revised method section.

2.What will be the benefit of the study? The introduction should specifically indicate the significance of the study as a separate paragraph. Moreover, this will inspire readers to take an interest in the subject matter. Please provide a structured ending statement in the introduction section that why this review article is important considering the field, its significance and novelty.

Our response: We reorganized and rewrote the last paragraph in the introduction section with consideration of these points.

3.I found minor issue, many typos and grammatical errors are seen in the paper. There are grammatical mistakes and typographical errors in the manuscript. The author should recheck this manuscript carefully and remove all such errors.

Our response: I am sorry to hear that. We hired a professional editor for the submitted version of our manuscript, but we have gone through the manuscript again and found a few errors, which have been corrected in the revised manuscript.

4.Future directions and future implications should be described in a clear manner with strong conclusion.

Our response: We have included this in the conclusion section.
