# Supplementary material for: Fucosyltransferase 8 (FUT8) and core fucose expression in oxidative stress response
Source: PLoS One. 2023 Feb 13;18(2):e0281516. doi: 10.1371/journal.pone.0281516 (PMC9924996; doi:10.1371/journal.pone.0281516)
Supplement: Attachment — Submitted filename: Response to Reviewer.docx [file pone.0281516.s004.docx]

*Reviewer #1: The authors addressed the reviewers' concern properly. The reviewer encourages does not have any further comments. The reviewer expects that the authors reveal the mechanistic insights why the FUT8 expression is upregulated during oxidative stress conditions in the next work.*

We appreciate your constructive comments and suggestions that have improved our manuscript.

*Reviewer #2: In the present manuscript, authors reported the relationship between changes in glycosylation and oxidative stress by using GlycoMaple. Unfortunately, this reviewer believes that the present data do not support the conclusions. The changes in core fucosylation should be confirmed by mass spectrometry, since the UEA-I lectin used in the present study is not specific for core fucosylation. Therefore, the present version of the study is not suitable for publication in this journal.*

Since we agree with you that lectin UEA-I does not label only the core fucose, we had already added the following description at the end of the Results section in the original version of our manuscript: The cell line used in this study expressed only Fut 1, 2, 8, 10, and 11 under the conditions tested, and only Fut 8 showed an increase with the drug treatments. Since fucosyltransferases other than Fut 8 were not expressed or were not increased with the drug treatment, we concluded that the increase in reactivity to UEA-I was a result of the increase in Fut 8 expression.

*Reviewer #3: The authors demonstrated that the treatment with 5H4PB and SFN upregulated FUT8 gene and induced its product, core fucose structure. This is a good example to examine the glycan structure by using the in silico glycan structure prediction tool GlycoMaple. However, the current version is too preliminary for the publication.*

* This study lacks positive control experiments for oxidative stress responses. The authors should reanalyze with H2O2 to conclude the upregulation of the FUT8 gene by the oxidative stress response.*

We found two datasets (NCBI BioProject: PRJNA200279, PRJNA252456) that included HaCaT cells exposed to H_2_O_2_ and analyzed them. We are reluctant to include these results in the manuscript because they are not consistent with the main objective of our study, which was to investigate the induction of the antioxidant response. Specifically, the expression of phase II antioxidant enzymes, the key signature of the antioxidant response, including NQO1 and GCLC, did not show an increase in cells treated with H_2_O_2_, suggesting that H_2_O_2_ treatment may not induce the desired response.

* Why did the authors analyze HaCaT cells? The authors may have a reasonable reason for this experiment.*

As stated in the Introduction section of the original manuscript, it is believed that the antioxidant response in the skin, particularly in keratinocytes, which is the source of the HaCaT cell line used in this study, is associated with skin sensitization, allergy, and dermatitis. We believed that identifying a marker that could detect early symptoms of such conditions could contribute to the diagnostic process.

* In the flow cytometric analysis in Figure 3, the induction of core fucose structure is significant but very weak. This flow cytometric analysis lacks staining controls. Lectin blotting is another good way to see core fucose structure. LCA or PhoSL would be much better lectin to identify core fucose.*

Following your suggestion, we performed Lectin UEA-I blotting as shown below. The drug treatment increased the UEA-I signal approximately 1.3-fold. The signal was normalized by anti-tubulin Western blotting. Lanes 1 and 2 show control and drug treated, respectively (left panel). We also performed FACS analysis using LCA as shown in the right panel. After the drug treatment, the LCA signal increased 2.8-fold, confirming the increased core fucose structure by the drug treatment.

* The authors also found a strong downregulation of the MGAT3 gene in Figure 1. How about bisecting GlcNAc structure?*

The halving of bisecting GlcNAc detected by lectin WGA (shown below) is consistent with the decrease in MGAT3 expression caused by the drug treatment (Figure 1), as expected. However, the focus of the current study is on Fut8, so this point is not included in this manuscript.

*The authors should select more suitable lectins for analyzing core fucose.*

We agree with you that the lectin UEA-I may not be ideal for core fucose labeling. However, we believe that our use of UEA-I is appropriate for the reasons described at the end of the Results section of the original version of our manuscript: The cell line used in this study expressed only Fut 1, 2, 8, 10, and 11 under the conditions tested, and only Fut 8 showed an increase with the drug treatments. Since fucosyltransferases other than Fut 8 were not expressed or were not increased with drug treatment, we concluded that the increase in reactivity to UEA-I was a result of the increase in Fut 8 expression.

* In the Discussion part, do not repeat the description mentioned in the Result part. Instead of it, the authors should discuss the relationship between glycan and oxidative stress response more deeply.*

* The authors mentioned that glycan changes by oxidative stress is the first report but it is not true. The following papers have been already published.*

We sincerely appreciate your bringing these references to our attention. We have now included them in the revised manuscript and also added and reorganized the discussion based on them.

Journal Requirements:

1. *Please ensure that your manuscript meets PLOS ONE's style requirements, including those for file naming. The PLOS ONE style templates can be found at https://journals.plos.org/plosone/s/file?id=wjVg/PLOSOne_formatting_sample_main_body.pdf and*

[*https://journals.plos.org/plosone/s/file?id=ba62/PLOSOne_formatting_sample_title_authors_affiliations.pdf*](https://journals.plos.org/plosone/s/file?id=ba62/PLOSOne_formatting_sample_title_authors_affiliations.pdf)

We have made the necessary adjustments to the best of our ability. Please let us know if any further corrections are needed.

2. *We note that you have stated that you will provide repository information for your data at acceptance. Should your manuscript be accepted for publication, we will hold it until you provide the relevant accession numbers or DOIs necessary to access your data. If you wish to make changes to your Data Availability statement, please describe these changes in your cover letter and we will update your Data Availability statement to reflect the information you provide.*

It’s available at https://www.ncbi.nlm.nih.gov/bioproject/897738

3. *We note that you have included the phrase “data not shown” in your manuscript. Unfortunately, this does not meet our data sharing requirements. PLOS does not permit references to inaccessible data. We require that authors provide all relevant data within the paper, Supporting Information files, or in an acceptable, public repository. Please add a citation to support this phrase or upload the data that corresponds with these findings to a stable repository (such as Figshare or Dryad) and provide and URLs, DOIs, or accession numbers that may be used to access these data. Or, if the data are not a core part of the research being presented in your study, we ask that you remove the phrase that refers to these data.*

We decided not to include the related information, as it was deemed unnecessary for the understanding of the manuscript.
